# African Swine Fever in Wild Boar in Europe—A Review

**DOI:** 10.3390/v13091717

**Published:** 2021-08-30

**Authors:** Carola Sauter-Louis, Franz J. Conraths, Carolina Probst, Ulrike Blohm, Katja Schulz, Julia Sehl, Melina Fischer, Jan Hendrik Forth, Laura Zani, Klaus Depner, Thomas C. Mettenleiter, Martin Beer, Sandra Blome

**Affiliations:** 1 Friedrich-Loeffler-Institut, Federal Research Institute for Animal Health, Institute of Epidemiology, Südufer 10, 17493 Greifswald-Insel Riems, Germany; Franz.Conraths@fli.de (F.J.C.); Carolina.Probst@bmz.bund.de (C.P.); Katja.Schulz@fli.de (K.S.); 2 Friedrich-Loeffler-Institut, Federal Research Institute for Animal Health, Institute of Immunology, Südufer 10, 17493 Greifswald-Insel Riems, Germany; Ulrike.Blohm@fli.de; 3 Department of Experimental Animal Facilities and Biorisk Management, Friedrich-Loeffler-Institut, Federal Research Institute for Animal Health, Südufer 10, 17493 Greifswald-Insel Riems, Germany; Julia.Sehl@fli.de; 4 Friedrich-Loeffler-Institut, Federal Research Institute for Animal Health, Institute of Diagnostic Virology, Südufer 10, 17493 Greifswald-Insel Riems, Germany; Melina.Fischer@fli.de (M.F.); JanHendrik.Forth@fli.de (J.H.F.); Martin.Beer@fli.de (M.B.); Sandra.Blome@fli.de (S.B.); 5 Friedrich-Loeffler-Institut, Federal Research Institute for Animal Health, Institute of International Animal Health/One Health, Südufer 10, 17493 Greifswald-Insel Riems, Germany; Laura.Zani@fli.de (L.Z.); Klaus.Depner@fli.de (K.D.); 6 Friedrich-Loeffler-Institut, Federal Research Institute for Animal Health, Südufer 10, 17493 Greifswald-Insel Riems, Germany; Thomas.Mettenleiter@fli.de

**Keywords:** African swine fever, disease control, wild boar, epidemiological course, diagnostic, clinical picture

## Abstract

The introduction of genotype II African swine fever (ASF) virus, presumably from Africa into Georgia in 2007, and its continuous spread through Europe and Asia as a panzootic disease of suids, continues to have a huge socio-economic impact. ASF is characterized by hemorrhagic fever leading to a high case/fatality ratio in pigs. In Europe, wild boar are especially affected. This review summarizes the currently available knowledge on ASF in wild boar in Europe. The current ASF panzootic is characterized by self-sustaining cycles of infection in the wild boar population. Spill-over and spill-back events occur from wild boar to domestic pigs and vice versa. The social structure of wild boar populations and the spatial behavior of the animals, a variety of ASF virus (ASFV) transmission mechanisms and persistence in the environment complicate the modeling of the disease. Control measures focus on the detection and removal of wild boar carcasses, in which ASFV can remain infectious for months. Further measures include the reduction in wild boar density and the limitation of wild boar movements through fences. Using these measures, the Czech Republic and Belgium succeeded in eliminating ASF in their territories, while the disease spread in others. So far, no vaccine is available to protect wild boar or domestic pigs reliably against ASF.

## 1. Introduction

African swine fever (ASF) is a devastating disease of domestic pigs and wild boar characterized by hemorrhagic fever that can be up to 100% lethal [1]. Despite its limited host range, its socio-economic impact is tremendous. It is therefore notifiable according to international and national regulations usually accompanied by strict control measures. Only members of the *Suidae* family are susceptible to ASF virus (ASFV), which has no zoonotic potential [2].

Over the last decade, ASF has changed from a regional disease of Sub-Saharan Africa to a considerable and tangible threat to pig husbandry, especially in Europe and Asia. After the introduction of genotype II into Georgia in 2007 and the subsequent spread in eastern Europe, the disease found a breeding ground in abundant wild boar populations [3,4,5,6,7,8]. Based on previous experience on the Iberian Peninsula and Sardinia with genotype I of ASFV, wild boar had so far not been considered a major and long-term reservoir for ASFV [9,10], and self-sustaining infectious cycles in wild boar were not anticipated at the beginning of the epidemic [11]. However, disease dynamics are different under the present conditions, particularly in north-eastern Europe, and long-lasting endemic cycles without any major involvement of domestic pigs were established in affected countries such as the Baltic States or Poland [3,4,5,7,12,13,14,15,16,17]. Despite the high virulence of ASFV and considerable case/fatality ratio among wild boar, these cycles remained self-sustaining in many affected countries over several years, while the Czech Republic and Belgium were successful in eliminating introductions of ASF in wild boar [18,19,20]. In Estonia and Latvia, there are indications that the ASF epidemic among wild boar is subsiding [5,13,14], although the emergence of a limited number of new cases in Estonia illustrates that control measures need to remain in place for a long time. 

The first step for assessing risks and planning control measures is gaining knowledge about critical factors in disease transmission and dynamics. In this context, this review summarizes the current knowledge on ASF in wild boar. 

## 2. Clinical Signs and Pathomorphological Lesions

### 2.1. Clinical Signs

Under field conditions, diseased wild boar often showed disorientation, e.g., by roaming at full daylight, staggering gait, lack of fear towards humans or dogs, and difficulties in breathing. Moribund animals and carcasses of wild boar that succumbed to infection were often found close to water bodies, where they seemed to seek a cold and moist environment, presumably as a reaction to fever [21].

Under experimental conditions, general clinical signs and pathomorphological lesions were quite similar between domestic pigs and wild boar [22,23,24]. However, for some virus strains with a slightly attenuated phenotype, e.g., “Estonia 2014”, clinical signs appeared more severe in wild boar [25,26]. 

**Figure 1:**
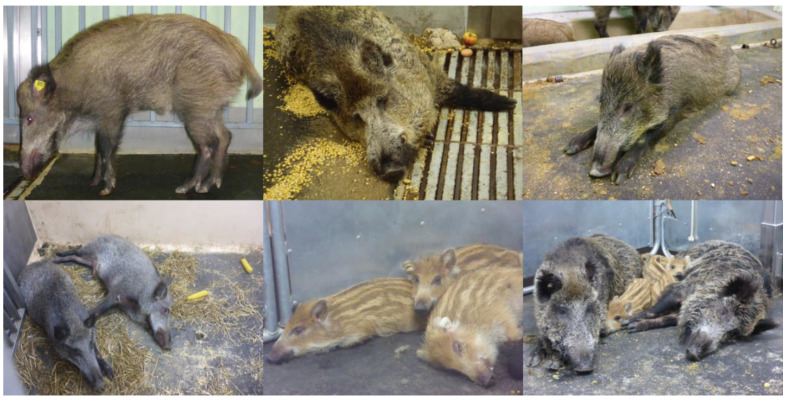
Clinical signs in wild boar of different age classes. Top row from left to right: hunched-up back and depression (**left**) in a sub-adult boar, severe depression and inappetence in a boar (**center**), severe but unspecific signs, respiratory problems in a sub-adult animal (**right**). Bottom from left to right: severe depression and moribund state in (sub-)adult females (**left**), piglets with high fever and reduced liveliness that later on recovered (**center**), and the same piglets and two adult females showing severe, unspecific signs (**right**).

Upon oro-nasal infection of wild boar with highly virulent ASFV strains, the first clinical signs were observed approximately four days post infection. Typical findings included high fever in all age classes, anorexia, depression, dullness, vomiting, diarrhea, reddening of the skin, respiratory disorders, and ataxia [22,23,25,27,28,29]. Severe hemorrhagic (epistaxis, bloody diarrhea, skin hemorrhages) and neurological signs were sometimes observed in the final stages of infection [27]. In the acute-lethal course of ASF, most animals died within 7 to 14 days post infection (dpi). However, some animals may survive longer or recover completely [28,30]. Typical findings are depicted in Figure 1.

### 2.2. Gross Pathological Findings

As common features after oral or oro-nasal infection, inoculated animals revealed swollen, enlarged, edematous and hemorrhagic lymph nodes, lung edema, which could be severe, varying renal cortical petechiae as well as hemorrhagic gastritis in some cases. More specifically, the hepatogastric and renal lymph nodes were generally more severely affected [23,26,28,29]. Occasional findings included a gall bladder wall edema [23,24,28], renal infarction [28], mild pulmonary consolidation [26], arthritis [28] and splenomegaly [23,29]. The latter is frequently mentioned as a characteristic feature of an ASF infection, but was only rarely observed after experimental infections, where humane endpoints are executed. 

Skin lesions were less common in wild boar than in domestic pigs. Nevertheless, hematomas or even subcutaneous petechiae have been described in animals inoculated by the intramuscular route using ASFV genotype I strains [31,32], but were less frequently observed after oro-nasal inoculation with genotype II strains [23].

Wild boar that were inoculated via the parenteral route further showed accumulations of fluid in the abdominal and thoracic cavity including hemohydroperitoneum [24,33] and pericardial or pleural effusion [31,32]. Moreover, hemorrhages in the intestinal tract or hemorrhagic enteritis have so far only been mentioned after intramuscular infection with strains of genotype I [24,33]. 

The development of lesions after ASFV infection has been observed in a single kinetic study investigating the above-mentioned Estonian ASFV strain showing a slightly attenuated phenotype, especially in domestic pigs [26]. Three wild boar were infected experimentally with ASFV “Estonia 2014”, sacrificed and necropsied on days 4, 7 and 10 post infection (p.i.). Lesions were generally mild to moderate. Swollen, hemorrhagic lymph nodes mainly affecting the renal and hepatogastric nodes were already observed on day 4 p.i. Renal petechiae occurred on day 10 p.i. in all wild boar, whereas only one animal developed a hemorrhagic gastritis on day 7 p.i. and another one pulmonary consolidation on day 10 p.i.

### 2.3. Histopathological Findings

Besides macroscopical lesions, histopathological changes were confined to lymph nodes, spleen, tonsil, liver, lung, kidney as well as to brain and male genitals, which appeared inconspicuous during necropsy. Regardless of the virulence of the virus strain used for infection, histopathology confirmed congestion and hemorrhages of affected lymph nodes and showed apoptosis of histiocytes and lymphocytes, referred to as lymphocytolysis [26,29,31]. In addition, the hemolysis of erythrocytes and the thickening of the connective stroma within lymph nodes have been described after infection with the Italian genotype I “Nemi” strain of ASFV [31]. Immunohistochemical antigen staining of lymph nodes showed positive myelomonocytic cells in wild boar infected with ASFV “Belgium 2018/1” and “Estonia 2014” [26,29]. Similar results were observed in lymphoid tissues such as spleen and tonsils, and included apoptosis of lymphocytes and histiocytes [26,29], congestion and hemorrhage [29] and thickening of splenic trabeculae and capsule [31]. Crypt necrosis and abscesses were also recorded. Viral antigen was present in splenic and tonsillar myelomonocytic cells as well as crypt epithelial cells within lesions in animals infected with the “Belgium 2018/1” and “Estonia 2014” ASFV strain [26,29]. Histopathological data from the liver only slightly differed among studies. Subcutaneous infection with the “Nemi” strain led to liver necrosis, periportal lymphocytic and granulocytic infiltration, as well as thickened septa, enlarged sinusoids and centrilobular veins [31]. Wild boar oro-nasally infected with the highly virulent “Belgium 2018/1” strain developed hepatitis, but also showed hepatic angiectasia and congestion [29], whereas both the degeneration and necrosis of Kupffer cells and lymphocytic inflammatory reaction were noticed after infection with the “Estonia 2014” strain [26]. In both groups, viral antigen was detectable in Kupffer cells, sinusoid endothelium and hepatocytes.

Irrespective of the virus strain, ASF infection invariably led to pulmonary inflammation. In detail, interstitial lymphohistiocytic infiltrates [26,29], lymphoid hyperplasia [29], alveolar edema, and hemorrhage [29,31] have been reported. In wild boar infected with the “Nemi” strain, the inflammatory pattern was different: alveoli and bronchi were filled with lymphocytes and cellular debris, and the bronchial epithelium was enlarged, indicating bronchopneumonia rather than interstitial pneumonia observed after infection with the Belgium and Estonian strain. Immunohistochemistry results obtained from the lung showed ASFV antigen-positive macrophages [26,29]. Kidney lesions including degeneration and necrosis of glomerular and tubular cells have only been reported after infection with the highly virulent strains “Nemi” and “Belgium 2018/1” [29,31]. Hemorrhages around vessels and between tubules were especially seen in Nemi strain-infected wild boar [31]. By contrast, infection with the Estonian strain showed rather mild interstitial lymphocytic infiltrates that were not necessarily associated with viral antigen [26]. Immunohistochemistry showed viral antigen in interstitial cells [26], macrophages, endothelium, glomerular and tubular epithelium [29]. 

An inflammation of the brain, which has not been further characterized, has so far been observed only once after experimental infection with the “Belgium 2018/1” strain [29]. Regardless of the presence of inflammation, viral antigen was detectable in macrophages [29], cerebral and cerebellar glia cells and choroid plexus epithelium [26,29] in wild boar infected with both the “Belgium 2018/1” and the “Estonia 2014” strain. Male reproductive organs have not yet been studied in detail, but were sampled in the study with the “Belgium 2018/1” strain [29]. While macroscopy revealed no changes, histopathological analysis of testis and epididymis showed hemorrhage and congestion, mild interstitial inflammation and single-cell necrosis as well as vasculopathy/vasculitis. Viral antigen was present in macrophages, endothelium, peritubular fibroblasts and to some extent in intraductal apoptotic cells. Accessory sex glands were normal, but revealed some positive macrophages. Although neither gross nor histologic lesions were detectable in bone marrow of ASFV “Estonia 2014”-infected wild boar [26] or salivary glands of animals infected with “ASFV Belgium 2018/1” [29], viral antigen was identified in myeloid cells, megakaryocytes and macrophages.

## 3. Immunology

As wild boar are part of ecosystems around the world, they are in contact with various pathogens [34]. Their pathogen reservoir is constantly changing and expanding through contact with livestock and humans [35]. Immunity is an important factor in this host–pathogen interaction, also with respect to the outcome of infection in the individual animal and the wild boar population as a whole [36]. Unfortunately, differences in the immunocompetence of the domestic pig and wild boar and thus differences in the immune response to ASFV infection have hardly been studied and are therefore not understood in great detail. 

Like in domestic pigs, the ASF virus replicates in wild boar mainly in monocytes and macrophages. However, in vitro studies by Giudice et al. [37], who infected monocytes and macrophages of wild and domestic pigs with the genotype I Sardinian ASFV field strain “22653/14”, showed differences between domestic pigs and wild boar. Monocytes of domestic pigs and wild boar were equally susceptible to the virus, but monocytes of wild boar did not respond by producing IL1α, IL1β, IL6, IL10, IL12, and IL18 after infection, while monocytes of domestic pigs did.

In ASFV-immunized domestic pigs, CD8+ T cells play a crucial role in immunity against ASFV. Depletion of CD8+ T cells from these pigs before challenge with highly virulent ASFV resulted in the loss of protection to the level of the non-immunized controls, while the animals with CD8+ T cells survived [38]. Such experiments are missing for wild boar. Recently, however, it could be shown that, after experimental infection with the highly virulent genotype II ASFV strain “Armenia08”, CD8+ T cells were activated in wild boar, in contrast to domestic pigs [39]. Interestingly, a loss of perforin expression on all cytotoxic T cells could also be observed in domestic pigs and wild boar on day 5 p.i. These results indicate a cytotoxic reaction of T lymphocytes in wild boar in response to a highly virulent ASFV infection, which is nevertheless not protective. After infection with the moderately virulent strain “Estonia2014”, a protective T cell immune response developed, illustrated by T cell activation and sustained proliferation, which apparently reduced mortality significantly [40]. Of particular interest is the more pronounced γδ T cell response in wild boar compared to domestic pigs, as measured by the increasing frequency of CD8+ cytotoxic γδ T cells in wild boar organs. The fact that wild boar became more severely ill despite this response can possibly be explained by a stronger expansion of regulatory T cells (Tregs), which may shorten the pro-inflammatory response [40]. These findings and interpretations are in accord with observations of a lymphohistiocytic interstitial pneumonia even after 10 days p.i., but only in domestic pigs infected with “Estonia2014”, which may indicate a prolonged pro-inflammatory response in domestic pigs in contrast to wild boar [26]. Sánchez-Cordón and co-workers conducted experiments in domestic pigs, which showed that Tregs might present a way of viral immune evasion as they were able to inhibit antiviral responses specifically [41]. These observations may indicate that this way of immune evasion is more prominent in wild boar than in domestic pigs.

The few wild boar that survive infection with ASFV usually seroconvert [25,28]. Depending on the ASFV strain and the route of infection, virus-specific antibodies can first be detected 11–20 days p.i. [42]. Experimental infections with highly virulent ASFV strains usually do not lead to measurable antibody titers in serum, because the animals reach the humane endpoint before mounting a measurable antibody response [23,43]. However, reports of PCR-negative, but seropositive, animals increase in the hunting bag of affected regions [44]. Among them are also young animals (<12 month) that seem to have survived the infection and possess ASFV-specific serum antibodies [45]. This age correlation has also been observed in experimental studies with ASFV “Estonia2014”: sera obtained from infected piglets contained ASFV-specific antibodies, while adults remained seronegative throughout the experiment [25]. A potential neutralizing effect of virus-specific antibodies is controversially discussed [46,47]. However, studies using domestic pigs revealed that antibodies play a role in protection [48]. On the other hand, it has recently been shown that immunization with the attenuated ASFV strain “OURT88/3” or the vaccine candidates “Benin∆MFG” and “HLJ/-18-7GD” failed to confer long-lasting protection in domestic pigs despite high antibody titers [41,49]. Similar studies with wild boar are lacking.

## 4. Epidemiology

### 4.1. Occurrence of ASFV in Europe

**Figure 2:**
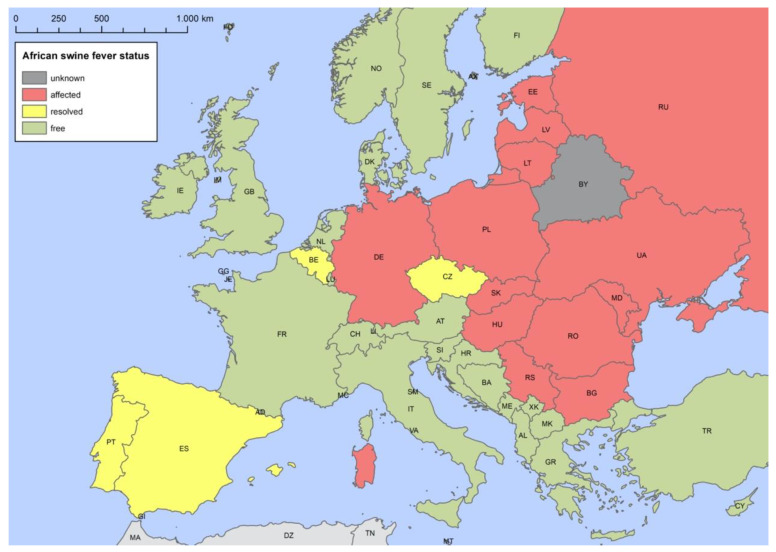
African swine fever status of the different European countries as of 4 August 2021.

Table 1 gives an overview of the introduction of ASF into different European countries in wild boar, and Figure 2 shows the current (as of 8 July 2021) status of ASF in the different European countries in wild boar. 

#### 4.1.1. Iberian Peninsula

ASF had been introduced into domestic pigs in Europe on several occasions since 1957. Historically, transmission to and long-term establishment in wild boar was only seen on the Iberian Peninsula (Portugal and Spain) and subsequently in Sardinia (Italy). 

Until 1981, only about 6% of the ASF outbreaks in domestic pigs were associated with direct contact to infected wild boar [67]. It was concluded that the disease would not have persisted in wild boar, once it had been eliminated from domestic pigs [10]. This was confirmed after the elimination of ASF from Spain and Portugal. In detail, a survey from 2006 to 2010 suggested that no wild boar in the affected areas were positive, thus indicating that the infection had not maintained itself in the wild boar population long term [68].

#### 4.1.2. Sardinia

ASF had entered Sardinia through contaminated food, similar to the introduction into Portugal. Since its first emergence in 1978, ASF remained endemic in Sardinia, with outbreaks occurring in domestic pigs and wild boar. The ASFV strain circulating in Sardinia belongs to genotype I [69]. In the south of Sardinia, ASF could be eliminated, while elimination attempts failed in the northern, central and eastern regions of the island for a long time [70,71], but the regions seem now to be close to becoming free from ASF [72]. 

Several risk factors for the persistence of ASF in Sardinia have been identified, such as the number of medium-sized farms, the presence of free-ranging pigs (particularly the local breed called “brado”) and the combination of the estimated wild boar density and the mean altitude above sea level [51,70,73,74]. 

Even after four decades of ASFV presence and circulation on the island, the prevalence in wild boar in Sardinia was found to be very low (1% with the 3rd quartile of 10% for seroprevalence for the time period 2011 to 2016) [71]. This is much lower than the recent prevalence in brado pigs, where antibodies against ASFV were found in more than 50% of the examined animals [74]. When a spatial-temporal analysis was performed, no clusters were identified in wild boar in Sardinia [75]. ASFV-positive wild boar were only found in endemic areas, where outbreaks in domestic pigs occurred [75,76]. Thus, similar to the situation in the Iberian Peninsula, ASF was apparently not able to establish an independent infection cycle in the wild boar population. It has been suggested that the disease would have spontaneously disappeared from the wild boar population, after elimination from domestic pigs [9]. Contacts between wild boar and infected free-ranging domestic pigs and repeated re-introductions into the wild boar population were postulated as necessary for the maintenance of the disease on the island [9,51,70,75,77]. A recent study [78] found direct and indirect interactions between free-ranging domestic pigs and wild boar in an ASF-endemic area of Sardinia, indicating that these contacts facilitated the transmission of ASF on the island. This was also confirmed in a retrospective study, which showed that spatial interactions between wild boar and brado pigs occurred close to pig farms [79].

#### 4.1.3. Russian Federation (RF)

ASF reached the RF through wild boar in November 2007, after introduction into Georgia and notification in the spring of that year, from where it also spread to Armenia, Azerbaijan, Abkhazia and South Ossetia [80,81]. The ASFV circulating in these areas was classified as a strain of genotype II [82]. The first cases of ASF in wild boar were detected in the North Caucasus region. In this area, which is considered to harbor a large wild boar population, transmission and persistence of ASFV in the wild boar population was observed [55,83]. Spill-over into domestic pigs was documented in 2008, when a traditional free-range pig holding was affected. Spill-back into the wild boar population occurred due to low biosecurity in the domestic pig holdings and through illegal disposal of infected material [55,84]. Until 2010, the disease spread within the southern territories of the RF epidemically, both in wild boar and in domestic pigs [11]. 

In the RF, human behavior played a major role in the spread of ASF [55]. Carcasses of infected pigs, hidden or unsafely disposed of by farmers, especially represented a constant source of introduction of ASFV into the wild boar population [55]. Infection in wild boar then could cause spill-back in domestic pigs due to low biosecurity [85]. In 2011, the spread of disease in the RF was estimated to be 350 km/year (N. Vlasov and D. Kolbasov, cited in [86]). 

Spatial analysis of data from the RF obtained between 2007 and 2013 revealed clusters of ASF in wild boar, domestic pigs and overlapping clusters [82]. Similar results were obtained for the time period 2017–2019 [87]. These studies indicate that the virus was able to persist in wild boar without constant reintroduction from domestic pigs in this region. This stands in sharp contrast to the situation that had been observed on the Iberian Peninsula and in Sardinia with the genotype I ASFV. 

#### 4.1.4. Baltic States, Poland and Germany

The situation in the Baltic states (Lithuania, Latvia, Estonia), Poland and Germany differs from the situation in the RF in that, in the former, the majority of ASF cases were detected in the wild boar population. 

The first introduction of ASFV genotype II occurred in January 2014, when Lithuania reported a case of ASF in wild boar near the border with Belarus. A few weeks later, the first ASF case was recorded in Poland in wild boar, also close to the Belarussian border. In June 2014, the first case of ASF in wild boar was detected in Latvia, again close to the border with Belarus [61]. Estonia followed with cases in wild boar that were detected in September 2014 in the southeast, close to the Latvian border, and in the northeast, close to the border with the RF. 

In Lithuania, the disease spread from the border to Belarus in a westerly direction. The number of infected wild boar increased substantially from 2014 to 2018 [88,89]. By 2019, about 86% of the area of Lithuania was affected by ASF [88,90]. 

In Latvia, the first area affected by ASF was the South-eastern border area with Belarus, followed by an introduction into wild boar in the north of Latvia, close to the border with Estonia, presumably through illegal disposal of offal into the forest [61]. In the meantime, nearly the entire territory of Latvia is affected by ASF [5]. 

In Estonia, ASF first emerged in two regions, one in the southern part, bordering Latvia, and one in the northeast, close to the border with the RF [4]. In the meantime, ASF has spread throughout Estonia (with the exception of the island of Hiiumaa). 

In Poland, until 2016, cases of ASF in wild boar were restricted to a belt of 1–10 km along the border with Belarus [8,91,92]. It is likely that repeated introductions from Belarus into Poland had occurred [15]. From 2014 to 2016, the cases showed a north-south distribution along the border, with an expansion of the affected area during 2017 [93]. In 2017 and 2018, the number of ASF-infected wild boar increased substantially [3,17]. At the same time, new areas were affected around Warsaw and in the north, close to the oblast of Kaliningrad (RF). In November 2019, a new region in western Poland became affected, first in wild boar and subsequently also in domestic pigs. 

Ten months later, in September 2020, the neighboring area across the border in Germany recorded the first ASF case in wild boar [6]. Both the vicinity of cases to the German–Polish border and their clustering suggest several independent incursions into Germany [94]. Together with sequence data of the complete viral genome obtained for several ASFV strains, this illustrates that there exists a transboundary epidemic affecting western Poland and Germany. 

In the Baltic states and Poland, the disease spread mainly through wild boar migration, but also through human activity. Transmissions over large distances (jumps) were obviously due to human activities (probably improper disposal of contaminated food waste), as described for several countries [61,95,96,97]. The local spread of ASF among wild boar was not as fast as originally predicted [98]. Depending on the wild boar density, the rate of local spread has been estimated to range between 1 and 2 km/month [99,100]. In Lithuania, it was calculated to be 5 km/month [88]. It should be noted that the spatial-temporal dynamics of ASF in wild boar could not exclusively be explained by the movements of wild boar, i.e., through dispersal and home range sizes [101]. 

Surveillance for ASF in wild boar is achieved by active and passive surveillance. Several studies have shown that passive surveillance of wild boar carcasses was superior to active surveillance in detecting ASF in wild boar [4,5,7,13,14,15,17,61]. Data on wild boar killed in traffic accidents are only sparse, and thus there has so far been no statistical evidence that the prevalence of ASF in road-killed wild boar is higher than in wild boar hunted healthy [102]. Nevertheless, wild boar killed in traffic accidents should be sampled in the interest of early ASF detection. At least during the early phases of an epidemic, the prevalence of ASFV-positive dead wild boar (usually determined by PCR, i.e., detection of ASFV genome) is much higher than the seroprevalence (i.e., animals with ASFV-specific antibodies) in hunted wild boar [4,7,14,90,102,103,104]. In Lithuania, the average prevalence of ASFV-positive wild boar found dead, as determined by PCR, was 65.7% (95% CI: 64.0–67.4%), while the serological prevalence in hunted animals (active surveillance) was only 0.45% (95% CI: 0.39–0.51%) with regional differences [89,90]. 

In all of these countries, the probability of finding an ASFV-positive animal was much higher in animals found dead in comparison to hunted animals with odds ratios between 69 and 193 [4,7,90]. 

During recent years, an increasing seroprevalence regarding ASFV of up to 7% was observed in all three countries of the Baltic states [5,14,44,90]. 

Several factors associated with the detection of the disease in wild boar have been investigated in these countries. Wild boar density was considered crucial for the spread of ASF. In the early years after Poland had become affected, cases of ASF in wild boar occurred mainly in areas with a wild boar population of more than 1 animal/km^2^. It was therefore concluded that reducing the wild boar density to less than this value could reduce the spread of the disease [15]. Polish data on ASF in wild boar between 2014 and 2016 revealed that the probability of finding ASFV-positive wild boar increased with wild boar population density and the proportion of forest in land cover [105]. 

Data from Estonia and Latvia revealed that young wild boar were more frequently ASFV-positive than older animals [4,14]. 

Another factor is seasonality. In the Baltic states and Poland, peaks in the detection of ASF in wild boar were observed during summer, but also in late winter (February and March) [8,61]. In Lithuania, the prevalence was higher in autumn than in spring [89] and high in winter [88]. In Estonia, a larger number of samples taken from wild boar found dead were recorded during winter [13]. In Latvia, however, no seasonality was detected in the occurrence of ASF in wild boar [7]. It has so far not been completely clarified whether the observed seasonal effects are due to a higher abundance of wild boar (in summer), increased hunting activity (in winter) or other, so far unknown factors. 

In contrast to observations made on the Iberian Peninsula, the numbers of recorded ASF cases in wild boar in the Baltic states and in Poland by far exceeded outbreaks registered in domestic pigs [93]. It is important to note that ASFV was obviously able to persist in the wild boar population in the Baltic states and Poland, regardless of the situation in domestic pigs. This stands in sharp contrast to previous predictions [9], which, however, were based on a different scenario.

#### 4.1.5. Czech Republic and Belgium

The ASF situation in the Czech Republic and in Belgium showed some similarities, but also differences, to that in the Baltic states and Poland. In contrast to multiple incursions into the wild boar population close to the border of neighboring countries, the Czech Republic and Belgium experienced focal or point introductions of ASFV into wild boar populations in regions more than 300 km away from known ASF-affected areas. It should be emphasized that in both countries only wild boar were affected. 

The first case of ASF in the Czech Republic was notified in June 2017 in two wild boar carcasses found near the local hospital in Zlín [20,106]. Presumably, the virus was introduced through human activity [20]. Genotyping identified the virus as similar to those variants that were found elsewhere in the eastern and south-eastern parts of the EU (Moldova, 2016; Ukraine, 2012 and 2015; Belarus, 2013) [106]. 

ASF remained limited to the district of Zlín [106], where a core area of about 58 km^2^ was defined [107]. In this core area, 71.7% of the sampled wild boar carcasses were ASFV-positive from June 2017 until April 2018 [106]. Until April 2018, when the last ASFV-positive wild boar was found, a total of 230 ASF cases were detected, of which 212 were in wild boar found dead and 18 in hunted animals [20,106]. This re-emphasizes the important role of passive surveillance in the early detection of ASF [107]. The last ASFV-seropositive wild boar were found in the core area in July and October 2018 [108]. One year after the detection of the last ASFV-positive wild boar, the Czech Republic submitted a self-declaration of the recovery of freedom from ASF to the World Organisation for Animal Health (OIE) [20]. 

Despite a high wild boar density in the Czech Republic, ASFV spread with a velocity of approximately 0.5 km/month, i.e., more slowly than in the Baltic States and Poland [109].

In the Czech Republic, ASFV-positive carcasses were found in larger distances from roads and forest edges than ASFV-negative carcasses [110]. In contrast to Estonia and Latvia, carcasses of adult animals were more frequently ASFV-positive than those of younger animals (75.4% versus 41.6%) [110].

Due to the fact that a single focal introduction had occurred into the Czech Republic, the basic reproduction number (R0) could be estimated at 1.95 for the first 29 days of the epidemic, i.e., each infected wild boar infected nearly two other wild boar [111]. Such calculations are neither available for the Baltic states nor for Poland. 

Similar to the Czech Republic, the introduction of ASF into wild boar in Belgium in 2018 was presumably caused by human activity. The first cases of ASF in wild boar were detected in September 2018 [65,112]. The virus was identified as genotype II and was found to be closely related to ASFV strains from Ukraine, Belarus, Estonia and European Russia [112,113,114]. 

Until March 2020, a total of 833 cases of ASF in wild boar had been reported by the Belgian Federal Agency for the Safety of the Food Chain [115,116]. In August 2019, the last fresh ASFV-positive wild boar carcass was detected; thereafter, all positive carcasses found were skeletonized (estimated postmortem interval at least 3–6 months) [115,116]. Since March 2020, no further ASFV-positive wild boar carcasses have been found. Belgium regained its OIE status “free from ASF” in December 2020.

Morelle et al. [21] investigated the deathbed choice of ASFV-positive wild boar carcasses from Belgium, Poland and the Czech Republic. They found that ASFV-positive carcasses were more likely to be found in cool and moist habitats [21]. 

For Belgium, an R0 of 1.65 has been calculated for the first 11 days in the second wave (starting at day 130 after detection of first case) [111] and a spreading velocity of 0.39 km/week [117]. The disease spread faster in forests than outside [117].

#### 4.1.6. Hungary, Romania, Slovak Republic and Serbia

Hungary, Romania, the Slovak Republic and Serbia reported cases of ASF in wild boar through the Animal Disease Notification System (ADNS) of the EU. However, no scientific publications on the epidemiology of ASF in wild boar in these countries are available. 

In April 2018, Hungary notified the first case of ASF in wild boar in the region of Heves, approximately 70 km south of the border to the Slovak Republic. Shortly afterwards, cases in wild boar were reported in the northeast of the country, close to the border with Ukraine. It was suspected that the introduction into the region of Heves was due to illegally imported contaminated pork products [118], whereas the cause of the introduction into the northeast of the country was assumed to be natural movements of wild boar from the Ukraine, where cases had been reported since 2017. Until the end of 2018, a total of 138 ASF cases in wild boar were notified. Until the end of 2020, nearly 6000 cases of ASF in wild boar had been notified, which were mostly localized in the northern and eastern regions of the country. 

Romania reported the first case of ASF in wild boar in May 2018, after outbreaks had occurred in domestic pigs in summer 2017 and from January 2018 onwards. The first reported case of ASF in wild boar was found in close vicinity to an outbreak of ASF in a small-holder pig farm. Until the end of 2020, nearly 1800 cases of ASF in wild boar were notified. It must be noted that the ASF situation in Romania is dominated by outbreaks in domestic pig holdings of all sizes, so that the infections in wild boar could actually represent spill-over events from the domestic pig sector [63].

Bulgaria reported its first cases of ASF in wild boar in the northeast of the country in October 2018, after an outbreak of ASF had occurred in domestic pigs in August 2018. Until the end of 2020, a total of 723 cases of ASF in wild boar were notified. The overall epidemiological situation in Bulgaria may not only resemble that in Romania, but is most probably linked to it.

In the Slovak Republic, the first ASF-cases in wild boar were reported in August 2019 in the south of the country, after outbreaks in domestic pigs had been reported close to the border with Hungary in July 2019. While in 2019, a total of 27 cases of ASF in wild boar have been reported, nearly 400 cases were reported in 2020. The affected area is close to a region in the north of Hungary, where ASF was first detected in wild boar in April 2018 and where the disease has since continued to spread.

Serbia reported its first outbreak of ASF in domestic pigs in August 2019 and the first cases of ASF in wild boar close to the border with Bulgaria in January 2020. Until the end of 2020, a total of 69 cases of ASF in wild boar were notified. 

### 4.2. Tenacity of ASFV

In the wild boar habitat, ASFV transmission may occur not only through direct contact with infected conspecifics, but also by indirect contact between susceptible wild boar and carcasses, excretions, food waste as well as contaminated environment, e.g., soil, water, grass or crops (wild boar–habitat cycle) [103,119]. Pepin et al. [120] estimated that over 50% of the transmission events in eastern Poland were related to indirect contact with an infectious carcass.

For indirect ASFV transmission within a wild boar population, virus survival in various matrices plays a crucial role [121], since this can last several months or even years (Table 2). Data concerning the tenacity of ASFV in wild boar organs and tissues that may persist for longer time intervals in the habitat, such as bones, muscle or skin, are scarce. While Fischer et al. [122] could not detect viable virus in bone marrow after one week at room temperature, Kovalenko et al. [123] were able to recover infectious virus from bone marrow stored at 6–8°C for more than six months. This difference could be due to the detection system, i.e., bioassay with the direct injection of pigs versus macrophage culture. Evidence suggests that muscle and skin/subcutaneous fat originating from wild boar represent long-term reservoirs for infectious ASFV, especially at low temperatures. On the other side, the stability of ASFV in urine and feces seems to be relatively low [122,124]. Virus survival in the soil underneath a carcass strongly depends on the soil type (sand vs. humus-rich soil) with survival times of less than three days to up to two weeks [125,126]. In contaminated water, infectious virus was detectable for at least 14 days [126], while a rapid decrease in infectivity was observed on dry field crops [127]. 

A study in Lithuania failed to find infectious virus in buried wild boar carcasses that had tested positive for ASFV before burial, but it could detect ASFV genome fragments in the surrounding soil [128].

**Table 2:** ASFV tenacity in different materials.

	Material	ASFV Stability	Method	Reference
Blood, organs and tissues	Defibrinated blood (RT)	140 days	In vivo assay	Montgomery 1921 [129]
	Blood (−20 °C)	6 years	In vivo assay	De Kock et al., 1940 [130]
	Preserved blood (4 °C)	18 months	In vivo assay	Plowright and Parker 1967 [131]
	Spleen suspension (−20 °C)	105 weeks	In vivo assay	Plowright and Parker 1967 [131]
	Spleen, kidney, lung (−20 °C)	112 days	Virus isolation in macrophages	Mazur-Panasiuk and Woźniakowski 2020 [126]
	Spleen, lung (4 °C)	56 days		
	Kidney (4 °C)	<28 days		
	Spleen, kidney (RT)	7 days		
	Bone marrow (6–8 °C)	>6 months	In vivo assay	Kovalenko et al., 1972 [132]
	Bone marrow, skin (−20 °C)	3 months	Virus isolation in macrophages	Fischer et al., 2020 [122]
	Bone marrow (4 °C)	1 month		
	Bone marrow, muscle (RT)	<7 days		
	Muscle (−20 °C)	>24 months		
	Muscle (4 °C)	3 months		
	Skin (4 °C)	6 months		
	Skin (RT)	3 months		
Feces and urine	Faeces (4 °C)	159 days	In vivo assay	Kovalenko et al., 1972 [132]
	Urine (4 °C)	60 days		
	Faeces, urine (4 °C)	5 days	Virus isolation in macrophages	Olesen et al., 2020 [133]
	Faeces (4 °C and 12 °C)	5 days	Virus isolation in macrophages	Davies et al., 2017 [124]
	Faeces (RT)	3 days		
	Urine (4 °C, 12 °C, RT)	5 days		
Soil, water, field crops and feed	Beach sand (RT)	14 days	Virus isolation in macrophages or cell culture	Carlson et al., 2020 [125]
	Yard soil (RT)	7 days		
	Swamp mud (RT)	3 days		
	Forest soil (RT)	0 days/none		
	Wet soil, leaf litter (4 °C & RT)	<3 days	Virus isolation in macrophages	Mazur-Panasiuk and Woźniakowski 2020 [126]
	Water (−20 °C, 4 °C, 23 °C, 37 °C)	>14 days		
	Water (−16 to −20 °C, 4–6 °C)	≥60 days	Virus isolation in macrophages	Sindryakova et al., 2016 [134]
	Water (RT)	50 days		
	Field crops (drying at RT)	<2h	Virus isolation in macrophages	Fischer et al., 2020 [127]
	Compound feed (−16 to −20 °C)	≥60 days	Virus isolation in macrophages	Sindryakova et al., 2016 [134]
	Compound feed (4-6 °C)	30 days		
	Compound feed (RT)	1 day		
	Compound feed (RT)	≥30 days	Virus isolation in macrophages, in vivo assay	Dee et al., 2018 [135]
	Soy oil cake (RT)	≥30 days		
	Compound feed (RT)	≥30 days	Virus isolation in macrophages	Stoian et al., 2019 [136]
	Soy oil cake (RT)	≥30 days		

### 4.3. Transmission of ASFV among Wild Boar

ASFV is the only known DNA arbovirus of vertebrates [2]. Its natural African vectors are soft ticks of the *Ornithodoros moubata* complex, which acquire the virus through blood-feeding on viremic hosts. They can harbor the virus for up to five years and transmit it vertically, horizontally, venereally and to susceptible suids during feeding [137]. In sub-Saharan Africa, the virus is transmitted in a sylvatic cycle from ticks to African warthogs (*Phacochoerus africanus*), which remain asymptomatic [138]. The ticks are nidicolous and live inside warthog caves and burrows, but they can also occur in the housings of domestic pigs [139]. In northern Europe, the occurrence of *Ornithodoros* soft ticks has never been reported. However, in southern Europe, different species of *Ornithodoros* soft ticks occur (e.g., *O. erraticus*, *O. maritimus*) and especially on the Iberian Peninsula they were responsible for ASFV infections in domestic pigs [140]. In the Caucasus region, *Ornithodoros* soft tick species have been documented to occur in some affected countries, such as Armenia, Russia and Georgia [141], but their involvement in ASF epidemiology seems unlikely, the more so since wild boar do not use caves, burrows or housings.

Other arthropods were repeatedly discussed as mechanical vectors. However, all arthropods tested so far, including hard tick species indigenous to Europe [142] as well as blowfly larvae [143], could not be incriminated. This is also in agreement with a study from Estonia, where various groups of blood-feeding arthropods were collected in ASFV-affected wild boar habitats but tested negative for the presence of the ASFV genome [144]. However, mechanical transmission on a laboratory scale was reported with the stable fly *(Stomoxys calcitrans*) [145] and two studies from Eastern Europe have reported the detection of a low amount of ASFV genome on insects collected on domestic pig outbreak sites [146,147]. 

Several incursions of ASFV into European wild boar populations have been mainly attributed to anthropogenic factors, especially to the dissemination of contaminated meat or meat products [92]. When ASFV was first introduced into Georgia in 2007, the first clinical cases were detected near the port of Poti [55]. Although the precise source of virus could not be identified, it has been suspected that ASFV was introduced via ships presumably from east Africa carrying contaminated meat or meat products, and free-ranging pigs acquired infection scavenging on the disposed waste [148]. Human failure to comply with biosecurity requirements has also been suspected to be the initial source of introduction into previously ASFV-free wild boar populations in Poland [149], Hungary [118], the Czech Republic [107] and Belgium [150]. This way of introduction is most likely not true for Germany. 

Considering the complexity of parameters, it is not surprising that the epidemiological patterns of ASF vary across different countries and regions. In Sardinia, where ASF has been present for 40 years in domestic pigs and wild boar, the persistence of the disease was related to traditional farming practices [70]. In the Russian Federation, the density of road networks, interactions with the domestic pig population and water bodies have been identified as the main risk factors for disease spread across the southern region of the country between 2007 and 2010 [151]. In the central part of the country, where a secondary endemic zone had formed since 2011, ASF outbreaks in wild boar were attributed to outbreaks in the domestic backyard sector [80]. 

On the other side, in current outbreaks, the wild boar density seems to be one of the most influential risk factors for the occurrence [152] and the transmission and persistence [153,154] of ASF. In Belgium, disease progression was related to the forest habitat and the wave front velocity was higher within forest areas than in non-forest areas [117]. In Latvia, the persistence of the infection was attributed to wild boar scavenging on carcasses of infected wild boar [61].

These observations have led to the hypothesis that ASF in wild boar is a habitat-borne disease [155] and to the description of the so-called wild boar–habitat cycle, which is self-sustaining and includes wild boar, their habitat and their carcasses [95,156].

To test this hypothesis, in a field study in Germany, 32 wild boar carcasses were exposed to study the behavior of wild boar towards their dead conspecifics. Wild boar were observed rooting in the decomposition islands, sniffing and poking on wild boar carcasses, and chewing on their skeletonized bones [157]. In the Czech Republic, during winter, even scavenging could be filmed [110]. Although it is not yet proven whether such types of interaction are sufficient for ASFV transmission, they support the hypothesis that transmission not only occurs through contact between susceptible and infectious animals, but also through contact between susceptible animals and contaminated material [103]. An age-structured mathematical model based on scenarios in Estonia and Spain further supported the assumption that transmission from infected carcasses to susceptible individuals is a key mechanism in producing disease outbreaks in wild boar [154].

Although it is known that ASFV has a high tenacity, details on the exact transmission routes in the wild boar–habitat cycle are still not known [158,159]. The fast localization and removal of carcasses is considered one of the most important disease control measures in affected regions [152]. However, depending on the weather, vegetation, field and light conditions, finding them can be difficult, and it is estimated that considerable numbers of infected carcasses are not found [12]. Estimating the postmortem interval of the first ASFV-positive carcasses in a previously ASF-free region can assist in estimating the time of disease introduction and the extent of the affected area. Yet, field studies have shown that the decomposition process of wild boar carcasses is highly variable and can take between a few days in summer and several months in winter [160,161].

Given the high stability of ASFV, concern has been expressed regarding scavengers that may spread infectious carcass material in the environment. However, evidence suggests that scavengers represent a minor risk factor for spreading ASFV, at least over large distances. On the contrary, they probably contribute to reducing local virus persistence by metabolizing infected carcasses [162]. However, the transfer of small amounts of ASFV-containing tissues over short distances, e.g., through birds, such as crows or birds of prey, could not be ruled out.

Other matrices that are regarded to play a potential role in the spread of ASFV among wild boar include oral-nasal excretions, blood, meat, offal, feces and urine, soil, insects, fomites, kitchen waste, as well as grass and other fresh vegetables contaminated by virus-containing matter [119]. 

The prediction of Bosch et al. [163] that the EU countries at the highest relative risk of ASF introduction by natural movements of wild boar were Romania, Slovakia, Finland, the Czech Republic and Germany has mostly been confirmed. However, the prediction that ASFV incursion into France was imminent due to the nation’s proximity to Belgium and the movement of wild boar across the border has so far not materialized [164]. 

Based on data from Poland 2014–2015, it was shown that wild boar contact rates are strongly constrained socially and spatially [101] and that wild boar movements are poor predictors of ASF dynamics in space and time [99]. This has led to the conclusion that the long- and medium-distance spread of ASF (i.e., >30 km) is unlikely to occur due to natural wild boar movements [165]. A recent EFSA report predicted that the natural median spread velocity of ASF in Belgium, the Czech Republic, Estonia, Hungary, Latvia, Lithuania and Poland due to wild boar movements was between 2.9 and 11.7 km/year [63].

### 4.4. Modeling

As there are many unknowns in the epidemiology of ASF in wild boar, several issues are addressed by modeling to gain a better understanding of the disease dynamics. 

Early during the current epidemic in eastern and south-eastern Europe, models focused on attempts to predict the spread of the disease and to assess the risk of introduction into countries that had not been affected so far. These assessments are of great interest for governments to plan preventive measures and prepare for outbreaks, but also for people directly affected by ASF, namely, pig holders and hunters. While there is sufficient data on domestic pigs in terms of abundance, transport routes and transmission routes, information on wild boar is scarce. Some of the early models on disease introduction therefore excluded wild boar. This was also done because it was believed that their expected contribution to the spread of ASF was less important compared to domestic pigs [67,166,167]. Yet, the epidemic situation as it developed since 2014 has shown that the spread of ASF in the wild boar populations in affected countries also matters. In particular, the introduction of ASF via wild boar movement, also across borders, has to be taken into account [163,168]. Several models have therefore been established that address the risk of ASF introduction into unaffected areas through trade and wild boar movements using statistical data fitting approaches [165,169]. 

The size of the wild boar population in a country is considered a risk factor in many models [153]. However, even in countries with small numbers of wild boar, such as Denmark, simulation models were used to assess the spread of ASF in wild boar and to minimize the risk of transmission to domestic pigs [153]. 

Other models focused on the presence of wild boar and habitat suitability for wild boar. Some of these model the suitability only, without including specific disease transmission risk [170]. Other models include habitat properties and suitability for wild boar in ASF transmission models. Halasa et al. (2019) applied an agent-based spatio-temporal model to the spread of ASF in wild boar in Denmark, using input parameters taken from the literature [153]. Another model combined the habitat suitability with an agent-based, spatially explicit simulation model that includes multi-source citizen science data on the presence of wild boar [171]. Croft et al. (2020) used a spatial individual-based model with data of a real landscape area in Britain for an isolated wild boar population [172]. 

Models have shown that the presence of suitable habitats for wild boar is a better predictive factor for the risk of ASF introduction than wild boar density [163,173,174]. Thus, models using environmental data proved to be a useful tool to estimate the risk of ASF introduction into naïve wild boar populations [154]. 

Several models [120,153,175] found that the presence of carcasses of infected wild boar could explain the transmission observed in real ASF epidemics. Pepin et al. [120] addressed the probability of transmitting ASFV from carcasses to live animals. Using data from eastern Poland and a spatially explicit mechanistic epidemiological model, they estimated that 53–66% of the transmission events may have been due to the presence of infected wild boar carcasses. They concluded that this carcass-based transmission is necessary to maintain the persistence of ASF in the wild boar population [120]. These data are corroborated by the long periods, over which ASFV can remain infectious in carcasses [110,128]. 

Another output of this model [120] was that carcass-based transmission becomes more important the lower the wild boar density is, as direct contacts are expected to become less frequent. The greater importance of transmission through carcasses of ASFV-infected wild boar as compared to direct transmission between living wild boar is also described by Lange and Thulke [175], who used a spatio-temporally explicit individual-based model [175,176]. This result can be explained by the long time wild boar carcasses remain in the environment, as compared to the relatively short remaining live-time of ASFV-infected animals [27]. Thus, the model showed the best fit to the observed spatio-temporal spreading pattern for the high accessibility of carcasses and a marginal chance of contact frequency of live wild boar to the carcasses [175]. This model was already used in 2015, after ASF occurred for the first time in the Baltic states and Poland, to evaluate different management options for eliminating ASF from the wild boar population [12]. A more recent model also suggested that environmental transmission, e.g., through carcasses of infected wild boar, is the main mechanism for outbreaks in the current epidemic in Europe [154]. These authors also propose that the role of wild boar in an ASF epidemic might be less significant in Spain, where temperatures are higher and wild boar carcasses will decompose faster, as compared to Estonia [154].

ASF in wild boar has proven to be extremely difficult to control [7]. Proposed measures, including massive depopulation in affected areas and the removal of wild boar carcasses, were considered not feasible or, as far as mass depopulation is concerned, unethical. On the other hand, measures that could have a long-term effect on wild boar, and thus on eliminating ASF, may take several years to become effective and have to be applied to larger areas. However, even then, the conclusion of different models is that only a combination of measures, including mass depopulation and carcass removal, are likely to be the most effective and feasible solution [12]. 

The comparison of control strategies was also the subject of a study conducted by Barongo et al. [177], who applied a model to free-ranging pig populations in resource-poor situations of Africa. They also used the model to determine optimal response times for control measures. 

Once control measures are effective, the aim of eliminating the disease from affected areas is often pursued. It is generally accepted that passive surveillance (the testing of all wild boar found dead or shot sick) is more suitable for early detection of the disease than active surveillance (the testing of hunted wild boar) [3,4,7,14,98,102,178]. However, this has been controversially discussed for later phases of the disease events. Therefore, Gervasi et al. [179] used a simulation model to evaluate the efficiency of active and passive surveillance for ASF in wild boar. Only in situations characterized by a low prevalence, a low wild boar population density and a high hunting rate was active surveillance superior to passive surveillance [179].

## 5. Diagnosis of ASF in Wild Boar

Controlling ASF in wild boar is highly dependent on early warning and thus on a rapid and reliable diagnosis [85]. Due to the international notification requirement, laboratory diagnosis is regulated in recommendations and legal requirements. Methods and protocols can be found in the OIE Manual of Diagnostic Tests and Vaccines for Terrestrial Animals [180] or, for the European Union, on the website of the European Union Reference Laboratory for ASF (https://asf-referencelab.info/asf/en/procedures-diagnosis/sops, accessed 24 August 2021). 

**Figure 3:**
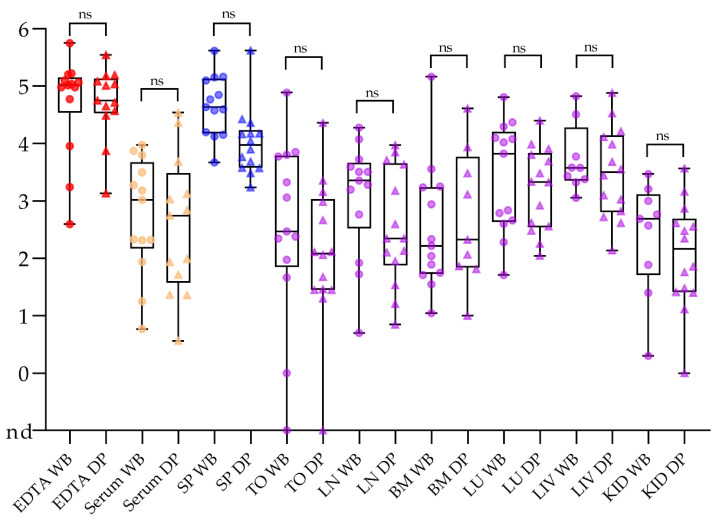
Comparison of sample matrices taken from wild boar (WB; dots) and domestic pigs (DP; triangles). The qPCR results are depicted as log10 genome copy numbers per run. Abbreviations: nd = not detected; SP = spleen, TO = tonsil; LN = lymph node; BM = bone marrow; LU = lung; LIV = liver; KID = kidney; ns = not significant in pairwise comparison. This figure was adapted from Pikalo et al. (2021) doi: 10.3390/pathogens10020177.

In general, reliable tools for the direct (pathogen) and indirect (antibody) diagnosis of ASF exist that work with appropriate samples from both domestic pigs and wild boar [181]. There is no difference in test performance or the suitability of matrices when testing high-quality samples from either domestic pigs or wild boar as depicted in Figure 3 [182].

### 5.1. Sample Matrices

Direct detection methods have priority to detect the disease in free areas at risk. For early detection, especially in the pre-clinical phase, EDTA blood and spleen samples are best suited using all types of real-time PCR methods [182,183]. In this phase, serum samples may yield false negative results, especially if pooling is applied [182]. Tonsil, lymph node, bone marrow, lung, liver and kidney samples are also suitable and mentioned in the respective manuals. 

In the wild boar context, it has to be kept in mind that the animals to be sampled are obviously sick or dead, so it can be assumed that a significant viral load is present in several organs and tissues [103]. In combination with the high stability of ASFV in wild boar carcasses [122,128], sampling can be facilitated by the use of pragmatic, alternative matrices that minimize the need to open and touch a rotten carcass. Over the last years, dry blood swabs have been validated for passive surveillance under experimental and limited field settings [182,183,184,185,186]. It could be demonstrated that these samples are suitable for the PCR detection of the ASF viral genome and, with a certain limitation, for the detection of antibodies. Recently, state-of-the art flocked swabs and inactivating transport buffers have been tested along the same lines with very promising results, especially for the transport buffer system [182]. Different bloody swabs have been put to practice in the Germany outbreak scenario since September 2020 and performed well [6]. Without the inactivating buffer system, virus isolation is possible over a period of roughly three days (unpublished results). However, for a full characterization of the disease situation, including the generation of virus strains for biological testing and sequencing, additional samples are helpful and should be encouraged. Apart from the above-described swabs, dried filter papers and FTA cards [187,188,189], fecal samples [142], oral, nasal and rectal swabs [190], meat juice [191], and different rope-based options [192,193] have been assessed as alternative, partly non-invasive matrices. Further matrices such as ear punches have been discussed and showed general suitability for diseased wild boar and domestic pigs [182]. If bloody material is no longer available, bone marrow from femur, humerus, jawbones, ribs, or sternum should be sent in for testing. Interestingly, this matrix performed much better if taken from skeletonized carcasses. In this case, it was superior for the use in shot-gun next-generation sequencing approaches (unpublished results and [6]).

Following the introduction of ASF into a new region, antibody detection becomes a valuable diagnostic tool to monitor disease evolution and potential changes in ASFV virulence. For the detection of antibodies, serum and plasma samples are the first choice mentioned in the respective manuals. However, filter papers [188], the above-mentioned swabs [184], meat juice, fecal material [194], and oropharyngeal fluids [195] could provide additional information if the collection of serum or plasma is not possible. 

In general, the choice of sample matrices should be embedded in the overall approach. 

### 5.2. Detection of ASF Virus, ASFV Antigen and Genome

Polymerase chain reaction protocols represent the first line of ASFV detection, and an increasing number of published protocols and fully validated test kits are available [196,197,198,199,200,201,202,203,204,205,206,207,208]. Regarding the later, commercial kits with reasonable pricing, the integration of internal control systems and the lack of need for extra consumables are key. As manufacturers are distributed around the globe, performance characteristics are not easy to compare. Within the European Union, some countries have an official licensing process and released products and batches underwent batch release. In Germany, for example, an updated list of licensed kits is included into the German Official collection of methods for notifiable diseases (https://www.fli.de/en/publications/amtliche-methodensammlung/, accessed 24 August 2021). All these tests have been tested at the German national reference laboratory (NRL) with a defined set of experimental samples representing different genotypes (mainly I and II), host species, matrices, and infection status [209]. The licensed kits are (as of March 2021): INgene q PPA (Ingenasa, Madrid, Spain), virotype ASFV and virotype ASFV 2.0 (Indical Bioscience, Leipzig, Germany), ID Gene ASF Duplex (IDvet, Grabels, France), RealPCR ASFV (IDEXX, Westbrook, ME, USA), SwineFever combi (gerbion, Kornwestheim, Germany), virella ASFV seqc (gerbion, Kornwestheim, Germany), ViroReal Kit ASF Virus (Ingenetix, Vienna, Austria), Kylt ASF Real-Time PCR (Anicon, Emstek, Germany), VetMAX African Swine Fever Virus Detection Kit (Thermo Fisher Scientific, Waltham, MA, USA), and the ADIAVET ASFV FAST TIME Kit (BioX, Rochefort, Belgium). Some of these commercial PCR tests and three routinely used automated extraction methods have been compared at the German NRL in more detail (Schlottau, unpublished). Nucleic acids were extracted from wild boar and domestic pig samples using the NucleoMag^®^ VET (Macherey–Nagel, Düren, Germany), MagAttract Virus M48 (Qiagen, Hilden, Germany), and MagMAX™ CORE (Thermo Fisher Scientific, workflow C) kits on the KingFisher extraction platform (Thermo Fisher Scientific, Waltham, MA, USA) according to the manufacturer’s instructions. In general, all kits were suitable and yielded reliable results in downstream applications. Recently, data were published on the comparison of seven commercially available PCR kits and three polymerase reaction mixes [210]. Here, the following kits were included: virotype ASFV 2.0 PCR kit, (Indical Bioscience, Leipzig, Germany), Adiavet ASFV Fast Time (Adiagen, Rochefort, Belgium), Bio-T kit ASFV (Biosellal, Dardilly, France), VetMax ASFV Detection kit (Thermo Fisher Scientific, Waltham, MA, USA), RealPCR ASFV DNA Test, (IDEXX, Westbrook, ME, USA), VetAlert ASF PCR Test Kit (Tetracore, Rockeville, MD, USA), and the ID Gene™ African Swine Fever Duplex (IDvet, Grabels, France). In brief, the diagnostic sensitivity and specificity was tested on 300 well-characterized wild boar samples collected in Belgium during the 2018–2019 outbreak. This study confirmed that all commercial kits and two out of three Taq polymerases (AgPath-ID™ One-Step RT-PCR Reagents, Applied Biosystems, Waltham, MA, USA, and TaqPathTM 1-Step Multiplex Master Mix, Thermo Fisher Scientific, Waltham, MA, USA) are suitable for ASFV detection in diagnostic laboratories. This is in line with the experience described above and confirms the suitability of commercial kits for a rapid and user-friendly ASFV diagnosis. Apart from traditional gel-based and real-time PCR assays, different alternatives including isothermal amplification methods have been designed and evaluated [205,211,212]. So far, they did not replace TaqMan-based real-time PCR protocols. 

Virus isolation is the gold-standard confirmatory test and required to obtain isolates for further characterization. Due to biosafety considerations and the requirement for susceptible cultures of primary cells, this technique is usually only applied in reference laboratories according to standard protocols [213]. Routinely, hemadsorption [214] is used as readout (for hemadsorbing strains).

Under resource-limited settings, the use of antigen ELISAs could be an option. They are commercially available, but lack sensitivity [181,215]. The same is true for antigen lateral flow assays, which showed some promising results in experimental settings [182,216], but would failed to detect most confirmed ASF cases from Germany (P. Deutschmann, personal communication).

Screening for ASFV-specific antibodies is usually done with ELISA-based methods. Again, several in-house and commercial tests are available. Most widely used within the European Union are probably the INGEZIM PPA COMPAC (Ingenasa), the ID Screen^®^ African Swine Fever Indirect (IDvet), and the ID Screen African Swine Fever Competition test (IDvet). These test systems use different antigens and they may therefore be used in parallel. Internationally, there are various test systems, whose performance is not easy to assess. It has to be noted that poor-quality serum samples (as they are often obtained from wild boar, particularly animals that were found dead) may affect test specificity. For this reason, confirmatory testing is recommended [181]. This can be achieved using indirect immunoperoxidase tests or immunoblotting.

## 6. ASF Control in Wild Boar

ASF is explicitly listed in Article 5 of the Animal Health Law of the EU (Regulation 2016/429) and affected Member States of the EU have to implement the respective prevention and control measures. The measures set a minimum standard, and national authorities can implement additional and stricter measures if necessary. 

In several studies, the presence of ASF-infected wild boar in close vicinity to domestic pig holdings is described as a main risk factor for ASF outbreaks [55,61,217,218]. Thus, successful ASF control in wild boar is crucial to protect the domestic pig sector from spill-over incidents. In contrast to ASF control in domestic pigs, which is achieved by depopulation of the farm and further measures that include cleansing and disinfection of the affected premises, movement restrictions, establishing restricted zones, etc., the control of ASF in wild boar is challenging [155,219]. There is no standard control strategy that can be applied everywhere in the same way. Moreover, there is no vaccine available currently. Suitable measures must be selected from a variety of options and need to be adapted to the specific epidemiological situation, as well as to environmental and social factors [220].

The prevention of ASF introduction into a non-infected area is the preferred option. Two different scenarios have been described for the introduction of ASFV into a naïve wild boar population [119]: (i) ASF-infected wild boar may introduce the virus in a neighboring region or country by cross-border migration. This has probably been the route of introduction into several European countries [4,6,61,91]. (ii) Due to the high tenacity of ASFV in raw meat products and the environment [122,126,129,130,131,221], the virus can also be transmitted indirectly by human activity and thus ‘jump’ over large distances, as observed in the Czech Republic and Belgium [106,107,222]. Since the location, where ASF emerges in the latter scenario, is hardly predictable, only rather general preventive measures can be applied. These usually aim at raising awareness, informing people about the risk of introducing ASFV with pork, pork products, hunting trophies, vehicles, clothes, shoes, equipment, etc., about cleansing and disinfection measures and the safe disposal of waste that may contain ASFV [119,223]. 

The role of the wild boar density in disease transmission and spread is highly disputed. However, there seems to be consensus among experts that a reduction in wild boar density decreases the risk of ASFV introduction and spread [224,225]. Efforts to reduce the population density usually imply increased hunting efforts, targeted hunting to decrease the number of reproductive females, and the trapping of wild boar followed by culling. Moreover, the use of supporting tools for hunting such as silencers or night vision is discussed and applied in some affected areas [225]. Population reduction through measures such as fertility control, poisoning or involving snipers are not only difficult to implement, but may also be legally and ethically contentious [224,225]. Building fences to protect an ASF-free region against the immigration of ASF-infected wild boar has been attempted. However, wild boar may overcome fences or find gaps or other ways to circumvent them. Limited acceptance by local people is expected [225], which may lead to constant damage or even robbery of the fences and continued efforts to repair, maintain or replace them. 

If the efforts to prevent ASFV introduction fail, the choice of control measures mainly depends on the route of introduction and the stage of the epidemic. In any case, early detection is vital. Due to the high case/fatality ratio of ASF, active carcass searches, i.e., enhanced passive surveillance, constitute one of the measures that need to be implemented as soon as possible [4,7,119,224]. Carcasses of wild boar that died of ASF contain large amounts of infectious virus and therefore represent a source of direct and indirect transmission [95,157,162,226]. Removing carcasses of potentially infected wild boar from the environment may thus help to minimize the risk of disease spread and maintenance.

After a single introduction event, successful elimination of ASF from wild boar populations has been demonstrated in the Czech Republic and Belgium [20,227]. Control measures were implemented in different zones around the affected area. Accordingly, fences or systems of layered fences (in Belgium and France, currently also applied in Germany) were built to avoid disease spread. Furthermore, hunting and feeding was banned within the inner zones, at least in the early phase of the epidemic, whereas in the outer areas hunting was intensified [119]. These control measures were implemented at different intensities for at least 10 or 14 months, respectively, before these countries submitted their self-declaration of freedom of ASF to the OIE. Fencing may also be applied to prevent the migration of potentially infected wild boar across national borders [94]. 

In regions where ASF was introduced by migrating wild boar, disease control is much more challenging. If there is constant infection pressure, e.g., along a border, control measures have to be implemented in larger regions or in several areas simultaneously and over a longer period. While this may be feasible at the beginning of a new epidemic, the longer the disease persists, the more likely it is that involved stakeholders reach their limits. Their acceptance of the control measures and their willingness to support them may decrease [228,229,230]. ASF control is more difficult in such an endemic disease situation. Most strategies applied in these scenarios refer to hunting, including the ban of driven hunts or promoting targeted hunting, but also passive surveillance and the removal of carcasses from the environment are conducted. However, the effectiveness of these measures has not yet been unambiguously demonstrated [63,96]. In most of the countries that have been affected by ASF in wild boar for several years, a significant reduction in the population density has been observed [7,13,100]. However, the disease is still present in these countries, so they also suffer from the corresponding economic restrictions, even if the ASFV prevalence decreased and no or hardly any cases in domestic pigs occur [3,5,13,16]. In Latvia, for example, several control measures had been implemented (e.g., incentives, targeted hunting, usage of supporting tools), but none of them showed an immediate effect on ASF prevalence [7].

Although most experts agree on the importance of reducing the wild boar population, to minimize disease spread, the implementation of the necessary steps to achieve this goal currently appears to be unsuccessful [219]. Besides the need to have a variety of control options available and to adapt them to the local situation, communication with the involved stakeholders is crucial, not only during the epidemic, but already before, to ensure a good cooperation in case of a disease emergence. Several participatory studies showed that control measures, performing well in models or in theory, were useless without the support of relevant stakeholders [228,229,231].

Generally, in endemic situations, the effort to detect and remove wild boar carcasses has to be maintained. Moreover, the consequent and ongoing reduction in the wild boar population should be aimed for. Furthermore, the awareness of potentially affected stakeholders, such as hunters or farmers, should be kept up. To avoid virus introduction into domestic pig holdings, high-level biosecurity measures should become a matter of course and, ideally, controls of these should be ensured by regular inspections through the competent authority [232].

While the examples of the Czech Republic and Belgium show that it is in principle possible to eliminate ASF from wild boar populations, the control of ASF in wild boar remains a major challenge and there is no one-size-fits-all solution.

## 7. Conclusions

After its introduction into Georgia in 2007, ASFV genotype II has spread within Europe and Asia. In contrast to previous introductions observed in the 20th century, wild boar now play an important epidemiological role. The disease occurs in self-sustaining infection cycles in wild boar populations without the need for involvement of the tick vector or domestic pigs, while spill-over infections into domestic pigs occur. Control measures include the removal of wild boar carcasses from the environment, reduction in wild boar density and building fences to restrict or avoid wild boar movements. Yet, only two countries succeeded in eliminating ASF from their wild boar population. The disease continues to spread elsewhere. 

## Figures and Tables

**Table 1:** African swine fever in wild boar and domestic pigs in Europe.

Country	Region	First Occurrence of ASF in Wild Boar	Presumed or Proven Route of Introduction	Reference	Presumed Main Driver for ASF Epidemic in Wild Boar
Portugal		n.d.		Costard et al., 2009 [50]	Interaction of domestic and free-ranging pigs/*Ornithodoros* ticks
Spain		n.d.	Spread from Portugal		
Italy	Sardinia	n.d.	Pork products from Portugal	Mannelli et al., 1998 [51]	Interaction of domestic and free-ranging pigs
Georgia		n.d.	Waste from ships	Vepkhvadze et al., 2017 [52]	n.d.
Armenia	North	Oct 2010	Movement of infected pigs and wild boar from Georgia	FAO 2008 [53]; Markosyan et al., 2019 [54]	Interaction of free-ranging domestic pigs and wild pigs
Azerbaijan		n.d.	Pork products from Georgia	FAO, 2008 [53]	n.d.
Russian Federation	Northern Caucasus	Nov 2007	Wild boar movements from Georgia	Gogin et al., 2013 [55]; FAO, 2008 [53]	Wild boar/free-ranging pigs
Belarus		n.d.	n.d.	GF-TADs, 2015 [56]	n.d.
Ukraine	Lugansk (2014)	Jan 2014	Wild boar movements from Russian Federation, 2014	DEFRA, 2014 [57]	Interaction of domestic and free-ranging pigs or backyard holdings
Lithuania		Jan 2014	Wild boar movements from Belarus	State Food and Veterinary Service Lithuania, SCoPAFF, Feb. 2014 [58]	Wild boar
Poland	East	Feb 2014	Wild boar movements from Belarus	Wozniakowski et al., 2016 [8]	Wild boar
	Warsaw	Nov 2017	Human activity	General Vet. Inspectorate, Poand, SCoPAFF, Jan 2018 [59]	Wild boar
	North	Dec 2017	Wild boar movements from Kaliningrad, RF	n.d.	Wild boar
	West	Nov 2019	Human activity (?)	Mazur-Panasiuk et al., 2020 [60]	Wild boar
Latvia	East	June 2014	Wild boar movements from Belarus	Olsevskis et al., 2016 [61]	Wild boar
	North	July 2014	Illegal disposal of contaminated material		
	Madona	Aug 2014	Human activity (?)		
Estonia	South	Sep 2014	Wild boar movements from Latvia	Nurmoja et al., 2017 [4]	Wild boar
	North	Sep 2014	Wild boar movements from RF		
Moldova		May 2019		GF TADs, 2016 [62]	Outbreak in domestic pigs or wild boar movements
Czech Republic	Zlín	June 2017	Illegal disposal of food waste	OIE, 2019 [20]	Wild boar
Hungary		April 2018	Illegal disposal of food waste	EFSA, 2020 [63]	n.d.
Romania	Satu Mare	May 2018	Human activity	EFSA, 2020 [63]	Outbreaks in domestic pigs and pig holding structure
	Danube Delta	June 2018			
Bulgaria		August 2018	Human activity, wild boar movement (from Romania?)	Zani et al., 2019 [64]	Outbreak in domestic pigs or wild boar movements
Belgium	Wallonia	Sep 2018	Illegal disposal of food waste	Linden, 2019 [65]	Wild boar
Slovak Republic		Aug 2019	Wild boar movements from Hungary	EFSA, 2020 [63]	n.d.
Serbia		July 2019	Wild boar movements from Romania / Bulgaria	Reuters, 2020 [66]	n.d.
Germany	Brandenburg and Saxony (Polish Border)	Sept 2020	Wild boar movements from Poland	Sauter-Louis, et al., 2020 [6]	Wild boar

^1^ n.d.—no data.

## Data Availability

Not applicable.
